# Optimizing the Live Attenuated Influenza A Vaccine Backbone for High-Risk Patient Groups

**DOI:** 10.1128/jvi.00871-22

**Published:** 2022-10-03

**Authors:** João P. P. L. Bonifacio, Nathalia Williams, Laure Garnier, Stephanie Hugues, Mirco Schmolke, Beryl Mazel-Sanchez

**Affiliations:** a Department of Microbiology and Molecular Medicine, University of Geneva, Geneva, Switzerland; b Geneva Center for inflammation research (GCIR), University of Geneva, Geneva, Switzerland; University of North Carolina at Chapel Hill

**Keywords:** influenza, high-risk patient, live attenuated vaccine, optimization, vaccine

## Abstract

Together with inactivated influenza vaccines (IIV), live attenuated influenza vaccines (LAIV) are an important tool to prevent influenza A virus (IAV) illnesses in patients. LAIVs present the advantages to have a needle-free administration and to trigger a mucosal immune response. LAIV is approved for healthy 2- to 49-year old individuals. However, due to its replicative nature and higher rate of adverse events at-risk populations are excluded from the benefits of this vaccine. Using targeted mutagenesis, we modified the nonstructural protein 1 of the currently licensed LAIV in order to impair its ability to bind the host cellular protein CPSF30 and thus its ability to inhibit host mRNA poly-adenylation. We characterized our optimized LAIV (optiLAIV) in three different mouse models mimicking healthy and high-risk patients. Using a neonatal mouse model, we show faster clearance of our optimized vaccine compared to the licensed LAIV. Despite lower replication, optiLAIV equally protected mice against homosubtypic and hetesubtypic influenza strain challenges. We confirmed the safer profile of optiLAIV in Stat1^−/−^ mice (highly susceptible to viral infections) by showing no signs of morbidity compared to a 50% mortality rate observed following LAIV inoculation. Using a human nasal 3D tissue model, we showed an increased induction of ER stress-related genes following immunization with optiLAIV. Induction of ER stress was previously shown to improve antigen-specific immune responses and is proposed as the mechanism of action of the licensed adjuvant AS03. This study characterizes a safer LAIV candidate in two mouse models mimicking infants and severely immunocompromised patients and proposes a simple attenuation strategy that could broaden LAIV application and reduce influenza burden in high-risk populations.

**IMPORTANCE** Live attenuated influenza vaccine (LAIV) is a needle-free, mucosal vaccine approved for healthy 2- to 49-year old individuals. Its replicative nature and higher rate of adverse events excludes at-risk populations. We propose a strategy to improve LAIV safety and explore the possibility to expand its applications in children under 2-year old and immunocompromised patients. Using a neonatal mouse model, we show faster clearance of our optimized vaccine (optiLAIV) compared to the licensed LAIV. Despite lower replication, optiLAIV equally protected mice against influenza virus challenges. We confirmed the safer profile of optiLAIV in Stat1^−/−^ mice (highly susceptible to viral infections) by showing no signs of morbidity compared to a 50% mortality rate from LAIV. OptiLAIV could expand the applications of the current LAIV and help mitigate the burden of IAV in susceptible populations.

## INTRODUCTION

Influenza A viruses (IAV) are the etiological agents of one of the most significant respiratory tract infections, accounting for approximately 2% of all annual respiratory deaths (1). Hospitalizations and deaths occur mainly among high-risk groups, which include children, elderly, pregnant women and immunocompromised patients (2–4). One of the most effective ways to prevent influenza illnesses is vaccination. Three types of vaccines are available against IAV: recombinant influenza vaccines (RIV) and split-virus inactivated influenza vaccine (IIV), both injected intramuscularly, and a live-attenuated influenza vaccine (LAIV), in the form of a nasal spray. LAIV uses the genetic backbone of a temperature sensitive (ts), cold-adapted (ca), attenuated (att) influenza strain derived from A/Ann Arbor/6/1960. These features restrict vaccine virus replication to the upper respiratory tract at 33°C, thus preventing disease onset (5, 6). LAIV mimics a natural infection, which brings the advantage of triggering mucosal (7), cellular and cross-protective (8, 9) immune responses. Hence, LAIV vaccine effectiveness (VE) ranges from 40 to 69%, which is equivalent to the VE observed for IIVs (10–14). The nature of this vaccine, however, excludes key-populations from LAIV recommendation guidelines. The major concern being higher rates of fever, rhinorrhea and wheezing observed following LAIV administration in children younger than 2 years old. Uncontrolled replication of the vaccine virus and reversion to virulence in immunocompromised patients has been reported for other attenuated vaccines (15–18). Therefore, different strategies have been explored to improve the safety of the LAIV with a particular focus on the modulation of IAV nonstructural protein 1 (NS1) (19–21).

NS1 is a virulence factor that modulates the cellular antiviral response (22). In part this was attributed to its interference with host mRNA maturation by interacting with the 30 kDa subunit of the cleavage and polyadenylation specificity factor (CPSF30). CPSF30 is a key factor in the polyadenylation complex. In its absence, cellular pre-mRNAs are not polyadenylated and thus rapidly degraded leading to a reduced host cell protein production. The interaction between NS1 and CPSF30 is well described in the literature (23) with a crystal structure showing the amino acids responsible for it (24). While positions 103 and 106 are important to stabilize the interaction, positions 108, 125 and 189 were shown to antagonize the innate immune response by reducing antiviral genes expression (25, 26). We recently demonstrated that during IAV infection, NS1 binding to CPSF30 is also essential to limit the unfolded protein response (UPR) induced by excessive neuraminidase production in the endoplasmic reticulum (ER) (27). UPR is an intrinsic mechanism cells use to adjust the folding capacity of the ER in times of elevated glycoprotein production. Viruses often exploit the UPR to enhance their replication (28, 29). Recent findings describe a cross talk between UPR and innate immune responses with implications on viral clearance and induction of an antiviral state (30, 31). Moreover, UPR contributes to the adjuvant effect of AS03, a licensed adjuvant present in the formulation of the inactivated influenza vaccine Prepandrix (32).

In this study, we applied a targeted approach to mutate the NS1 protein of the LAIV backbone rendering it unable to bind the host factor CPSF30. The goal is to improve the safety profile of the existing LAIV and present a suitable vaccine candidate for a broader target population. We confirm that our approach would not reduce vaccine manufacture efficiency or production using standard models. We demonstrate a safer replication profile of our optimized vaccine (optiLAIV) in three relevant *in vivo* models representative of adult, infant and highly susceptible populations. OptiLAIV provided similar protection compared to the current LAIV. As a mechanism of action, we propose a self-adjuvanted effect through activation of the UPR, independent of the cellular interferon response.

## RESULTS

### Limiting NS1-CPSF30 binding ability does not impact vaccine virus growth.

We generated a LAIV parental strain encoding the genetic backbone (segments 1, 2, 3, 5, 7 and 8) of the cold-adapted influenza A/Ann Arbor/6/1960 (H2N2) and segments 4 and 6 (hemagglutinin and neuraminidase) from A/Puerto Rico/8/1934 (H1N1) as described previously (33). We refer to this 6 + 2 virus as LAIV. In a targeted approach, we abolished exclusively the ability of NS1 to interact with the cellular host factor CPSF30. We used targeted mutagenesis to introduce the following amino acid changes in the LAIV backbone: F103S, M106I, K124E, D144R and D189G. We then rescued the mutant virus by reverse genetics and refer to this vaccine candidate as optiLAIV.

**FIG 1:**
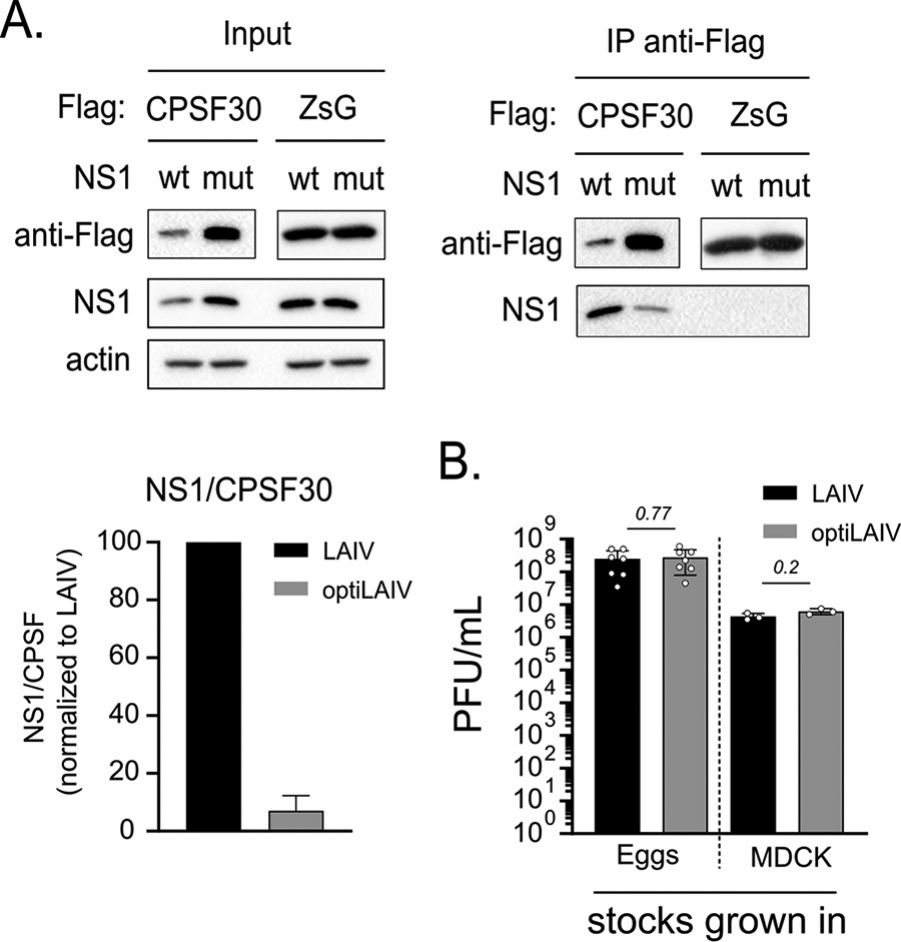
OptiLAIV NS1 binds to CPSF30 without compromising vaccine virus growth in eggs or MDCK cells. (A) 293T cells were transfected with pCAGGS.Flag-CPSF30 or pCAGGS.Flag-ZsGreen for 24 h prior to infection with either LAIV or optiLAIV at a multiplicity of infection (MOI) of 5. Cells were incubated for 16 h at 33°C and then lysed. Anti-Flag M2 affinity beads were used to immunoprecipitate (IP) the flag-tagged protein and its interactors. Precipitates were separated by SDS-PAGE and detected by Western blotting (WB) using the anti-flag antibody and the anti-NS1 antibody. The amount of NS1 protein that was bound to Flag-CPSF30 was quantified via a densiometric analysis (*n* = 2). (B) 10-day-old embryonated chicken eggs were infected with 100 PFU, and MDCK B cells were infected at a MOI of 0.1. After 46 h at 33°C, allantoic fluid and supernatant were collected. Viral titers were determined via plaque assay in MDCK cells at 33°C. Graphs represent the mean ± standard deviation (SD) of *n* = 3 to 7 repeats. Statistical significance was determined using a Mann-Whitney test.

To confirm that the five amino acids mutations introduced abrogate NS1-CPSF30 binding in context of infection, we overexpressed a flag-tagged CPSF30 protein in HEK 293T cells and superinfected those cells with either vaccine virus. Following immunoprecipitation of CPSF30, we successfully retrieved LAIV NS1. Compared to LAIV, optiLAIV NS1 coprecipitation was reduced by 80%, indicating reduced CPSF30-NS1 interaction (Fig. 1A). Enhanced attenuation of LAIV could result in deleterious replication defects, with unwanted consequences for large scale vaccine production (19, 21). We thus compared the replication of LAIV and optiLAIV in embryonated chicken eggs and MDCK cells, two relevant vaccine production models. Titers of LAIV and optiLAIV were similar in both production systems (Fig. 1B).

These results indicate that the targeted mutagenesis used to produce optiLAIV significantly impaired its NS1 protein ability to bind CPSF30 without compromising its potential for large-scale vaccine production.

### OptiLAIV is attenuated without compromising protection in adult mice.

**FIG 2:**
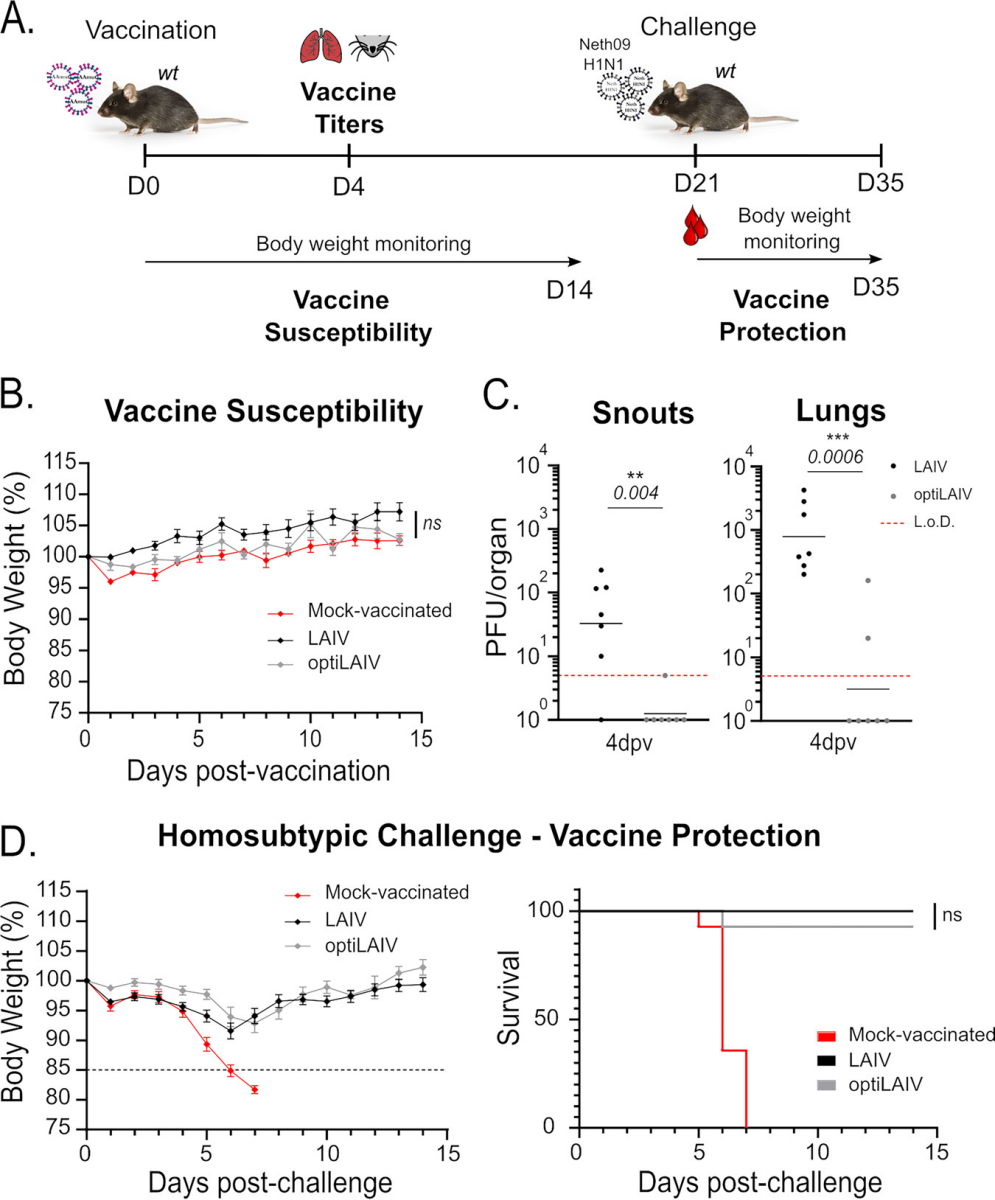
OptiLAIV is attenuated and protects adult mice against a homosubtypic/heterologous challenge. (A) Scheme of mouse immunization and challenge. (B) Female 8-week-old mice were vaccinated intranasally under anesthesia with 10^4^ PFU LAIV or optiLAIV in 25 μL PBS and body weight was monitored for 14 days postvaccination. (C) Female 8-week-old mice were vaccinated intranasally under anesthesia with 10^5^ PFU LAIV or optiLAIV in 25 μL and at 4 days postvaccination snouts (left panel) and lungs (right panel) were collected and vaccine viral titters were determined by plaque assay (*n* = 7 per group). (D) Female 8-week-old mice vaccinated with 10^4^ PFU of LAIV or optiLAIV were challenged at day 21 postvaccination with 20 PFU (10×LD_50_) of mouse adapted A/Netherlands/602/2009 (H1N1) in 20 μL PBS under light anesthesia (*n* = 14 per group). Body weight (left panel) and survival (right panel) were monitored for 14 days postchallenge. The statistical significances between LAIV group and optiLAIV group were determined using Mann-Whitney test in panel C; two-way ANOVA with the Geisser-Greenhouse correction and *post hoc* Dunn’s multiple-comparison test in panels B and D and Mantel-Cox test for survival curve. *P-values* are indicated in the figure. L.o.D. = limit of detection (5 PFU). Graphs are representative of 3 independent experiments and indicate geometric mean for panel C or mean ± SEM for panels B and D. Symbols represent data from individual mice for panels a and b. In D, black dotted line represents 15% body weight loss cutoff.

In order to demonstrate that our targeted NS1 mutagenesis strategy results in an attenuated phenotype *in vivo*, adult mice were vaccinated with a weight-adjusted dose of LAIV or optiLAIV according to recommendations for human adults (34) (Fig. 2A and Fig. S1). Then we monitored body weight as a general indication of well-being and viral titers in the upper and lower respiratory tract as an indication of viral fitness. In comparison to mock-vaccinated animals, neither vaccine virus caused alterations in body weight of adult mice (Fig. 2B), reflecting the already attenuated phenotype of the parental vaccine virus in immunocompetent hosts. However, we observed a clear difference in viral replication both in the upper and lower respiratory tract. Indeed, at 4 days postvaccination, we detected LAIV in both upper and lower respiratory tracts of vaccinated mice, while optiLAIV was absent in all snout samples and was present in only 2 out of 7 (28%) lung samples at viral loads close to the limit of detection (Fig. 2C).

The mutation introduced in the NS1 of the optiLAIV renders it less efficient in limiting host transcription (35, 36), which might result in undesired detrimental inflammatory responses. A hallmark of lung inflammation is the infiltration of immune cells into the lung tissue. Thus, we characterized the immuno-population of the respiratory tract of vaccinated mice at 4 days postvaccination. When comparing lungs and snouts of mice administered with PBS, we observed that, optiLAIVdoes not significantly changes the total of cell infiltrates or population composition compared to the LAIV (Fig. S2). When considering the optiLAIV as an alternative candidate for LAIV, the absence of viral load and comparable cellular infiltration in the respiratory tract argue in favor of a generally increased safety profile.

Since, previous studies associated lower replication fitness of different LAIV strains to lower immunogenicity and decreased vaccine effectiveness (37, 38), we evaluated if the protection elicited by optiLAIV was compromised compared to LAIV. 8 weeks-old mice were vaccinated intranasally with increasing doses of LAIV or optiLAIV. At 21 days postvaccination, the animals were challenged with influenza A/Netherlands/602/2009 (H1N1) strain, simulating a severely drifted virus based on HA sequences (homosubtypic/heterologous challenge). We monitored body weight (Fig. 2D – left panel) and survival (Fig. 2D – right panel) for 14 days after challenge. All animals were protected after a weight-adjusted dose for either vaccine (Fig. 2D). While we found a trend toward a longer recovery of the initial body weight (Fig. S1A) and a reduced protection with lower vaccine doses of optiLAIV (Fig. S1B), there was overall no statistically significant difference observed for the protective dose 50 (PD_50_) of both vaccines (Fig. S1C). In conclusion, compared to LAIV, our vaccine candidate presented impaired replication in the respiratory tract of adult mice, while still conferring similar protection against infection with a virus harboring a severely drifted HA.

### OptiLAIV is attenuated without compromising protection in neonatal mice.

**FIG 3:**
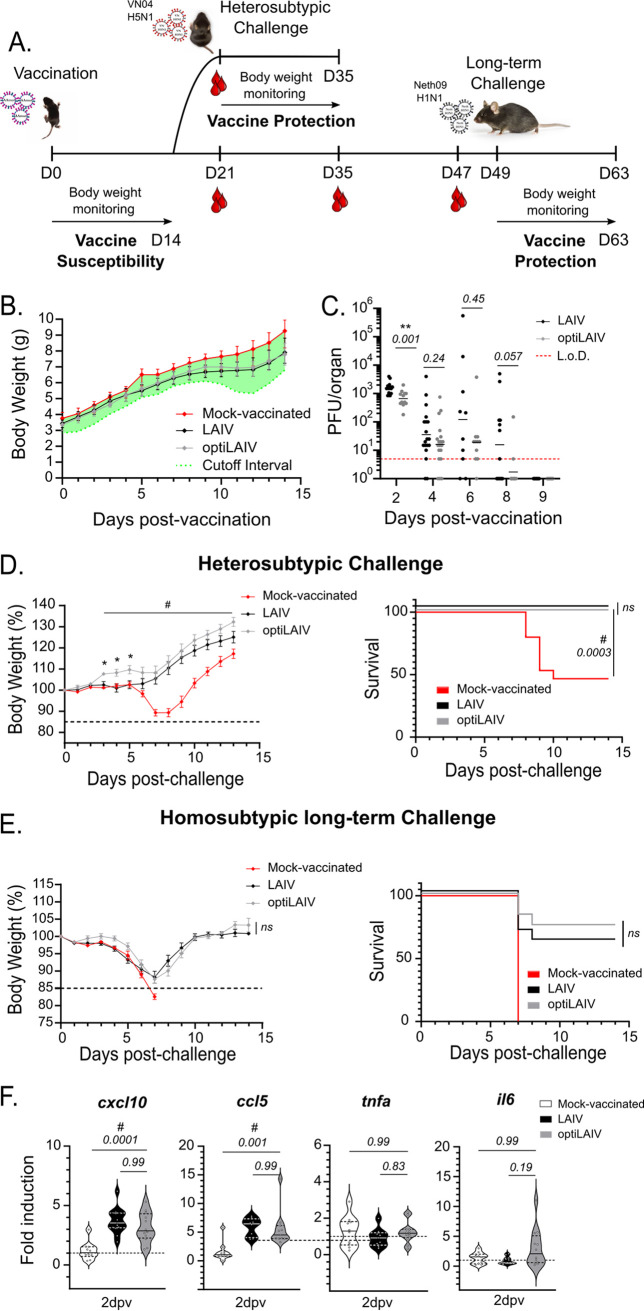
OptiLAIV is attenuated and protects neonatal mice against homosubtypic/heterologous challenge and heterosubtypic challenge. (A) Scheme of mouse immunization and challenge. (B) Seven-day-old mice were vaccinated intranasally with 10^4^ PFU LAIV or optiLAIV in 5 μL PBS and body weight was monitored for 14 days postvaccination. Cutoff was defined as twice the difference between the average weight of the nonvaccinated group and the lightest mouse of that group, calculated daily. (C) Seven-day-old mice were vaccinated intranasally with 10^5^ PFU of LAIV or optiLAIV in 5 μL PBS. At 2, 4, 6, 8 and 9 days postvaccination vaccine viral titters in the snouts were determined by plaque assay (*n* = 11 to 19 per group). (D) Seven-day-old mice vaccinated with 10^4^ PFU of LAIV or optiLAIV were challenged at day 21 postvaccination with 10^3^ PFU (20×LD_50_) of A/Vietnam/1203/2004 (H5N1) in 20 μL PBS under anesthesia (*n* = 11 per group). Body weight (left panel) and survival (right panel) were monitored for 14 days postchallenge. (E) Seven-day-old mice vaccinated with 10^4^ PFU of LAIV or optiLAIV were challenged at day 49 postvaccination with 20 PFU (10×LD_50_) of A/Netherlands/602/2009 (H1N1) in 20 μL PBS under anesthesia (*n* = 11 to 17 per group). Body weight (left panel) and survival (right panel) were monitored for 14 days postchallenge. (F) Seven-day-old mice (*n* = 11 to 12) were vaccinated intranasally with 10^5^ PFU of LAIV or optiLAIV in 5 μL PBS. At 2 days postvaccination snouts were harvested and RT-qPCR was performed from isolated RNA. The statistical significances between LAIV group and optiLAIV group were determined using multiple Mann-Whitney tests in panel C; two-way ANOVA with the Geisser-Greenhouse correction and *post hoc* Dunn’s multiple-comparison test in panels B, D and E and Mantel-Cox test for survival curves; Kruskal-Wallis test and *post hoc* Dunn’s multiple-comparison test in panel F. *P-values* are indicated in the figure. *, comparison between LAIV and optiLAIV; **^#^**, comparison between mock and optiLAIV. L.o.D. = limit of detection (5 PFU). Graphs are representative of 2 independent experiments and indicate geometric mean for panel C; mean ± SEM for panels B, D and E; mean ± SD for panel F. Symbols represent data from individual mice for panel C. In D and E, black dotted line represents 15% body weight loss cutoff.

An important target population outside the recommendation guidelines for LAIV administration are children under 2 years of age (39, 40). Indeed, clinical trials have reported higher incidence of adverse events, such as wheezing and coughing following vaccination (41, 42). To demonstrate an improved safety profile of optiLAIV in an animal model closer to this target population, we vaccinated neonatal mice (7 days old) with an adult dose of LAIV and optiLAIV. We then monitored body weight daily for 14 days and vaccine virus replication in the respiratory tract every other day up to 9 days after vaccination (Fig. 3A). Neither vaccine caused any significant alteration in body weight gain of neonatal mice (Fig. 3B), once again reflecting the already attenuated phenotype of LAIV. In line with the results obtained in adult mice, at 2 days postvaccination we observed lower viral titers in optiLAIV-vaccinated animals compared to LAIV in the upper respiratory tract (Fig. 3C). While at day 4 viral loads of both vaccines were reduced compared to day 2, we observed that at day 8 postvaccination, LAIV was still present in 7 out of 13 (53%) vaccinated animals. This was not the case for the optiLAIV group, where only 2 out of 12 (16%) vaccinated animals had detectable virus. After 9 days, both vaccine viruses were cleared from the upper respiratory tract. In lungs, we did not consistently detect virus after infection with either vaccine (data not shown), which is most likely a consequence of vaccine administration in the absence of anesthesia. We conclude from these data that optiLAIV reaches lower peak titers and is cleared faster from the airways of neonatal mice compared to LAIV.

The immune system of young children is in an immature state with compromised immune responses to vaccines (43). Thus, we asked if LAIV administered in our neonatal mouse model could elicit protection and if the attenuated phenotype of optiLAIV would impair this effect. To answer this question, we evaluated protection after a heterosubtypic challenge with A/Vietnam/2004 (H5N1) (Fig. 3D) or a homosubtypic/heterologous challenge with influenza A/Netherlands/09 (H1N1) (Fig. 3E). In both challenge models, significant protection was observed for both vaccines in a similar fashion (Fig. 3D and E – right panels). Unexpectedly, after heterosubtypic challenge, mock-vaccinated mice showed 50% survival after challenge with 20 adult LD_50_, nevertheless significant protection was still observed in vaccinated animals even with lower doses of both vaccines (Fig. S3A, B). After the homosubtypic/heterologous challenge, only the adult-dose of optiLAIV was protective compared to LAIV (Fig. 3E and Fig. S3C, D). However, there was no overall statistically significant difference observed for the protective dose 50 (PD_50_) of each vaccine (Fig. S3E). In conclusion, similar to LAIV, our vaccine candidate optiLAIV protects neonatal mice against both a homosubtypic and a heterosubtypic challenge.

Higher adverse reactions observed in young children might be explained by an exacerbated host response to a replicating virus (41, 44, 45). By unblocking host transcription and protein translation, optiLAIV could induce a damaging host response in young patients and worsen potential adverse reactions in this target population. Hence, we assessed the expression of two inflammatory genes implicated in a deleterious response in neonatal mice respiratory tract at 2 days postvaccination, when vaccine titers peaked (Fig. 3C). No significant expression of *Tnfa* and *Il6* was observed compared to mock-vaccinated animals (Fig. 3F). Furthermore, both vaccines induced expression of antiviral cytokines *Cxcl10* and *Ccl5* at similar levels. Overall, our data do not support a differential induction of innate immune or inflammatory genes by optiLAIV that could exacerbate any unwanted reactions compared to LAIV.

### OptiLAIV is attenuated and safer in Stat1^−/−^ adult mice.

**FIG 4:**
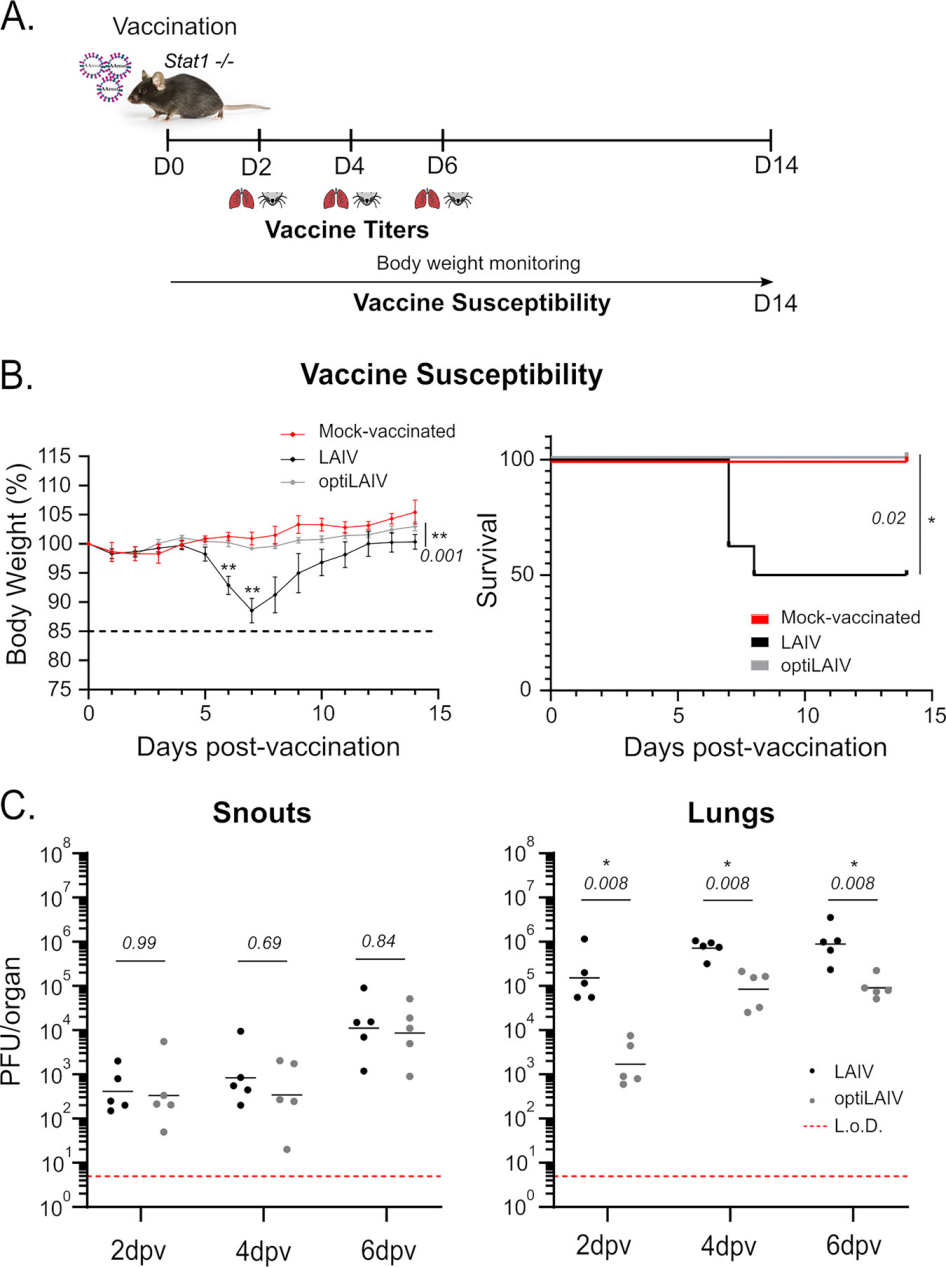
OptiLAIV is attenuated and safe in Stat1^−/−^ mice. (A) Scheme of mouse immunization. (B) 8-week-old Stat1^−/−^ mice were vaccinated intranasally under anesthesia with 10^4^ PFU LAIV or optiLAIV in 25 μL and body weight (left panel) and survival (right panel) were monitored for 14 days postvaccination (*n* = 4 mock-vaccinated group; *n* = 8 per vaccinated group). (C) 8-week-old Stat1^−/−^ mice were vaccinated intranasally under anesthesia with 10^5^ PFU LAIV or optiLAIV in 25 μL PBS. At 2, 4 and 6 days postvaccination snouts (left panel) and lungs (right panel) were collected and viral titers were determined by plaque assay (*n* = 5 per group). The statistical significances between LAIV group and optiLAIV group were determined using two-way ANOVA with the Geisser-Greenhouse correction and *post hoc* Dunn’s multiple-comparison test in panel B and Mantel-Cox test for survival curve; multiple Mann-Whitney tests in panel C;. *P-values* are indicated in the figure. *, comparison between LAIV and optiLAIV. L.o.D. = limit of detection (5 PFU/mL). Graphs are representative of 2 independent experiments and indicate mean ± SEM for panel B and geometric mean for panel C. Symbols represent data from individual mice for panel B. In C, black dotted line represents 15% body weight loss cutoff.

Due to its replicative nature, LAIV is not recommended for immunocompromised patients (39, 40) who might struggle to clear the vaccine virus. Further attenuation of this vaccine could be beneficial to this target population. To simulate a patient highly susceptible to viral infections, we used Stat1-deficient (Stat1^−/−^) adult mice, which are largely devoid of type I, II and III interferon responses and consequently lack key defense mechanisms to combat pathogen challenges. Stat1^−/−^ adult mice were vaccinated with an adult dose of LAIV or optiLAIV and we monitored body weight after vaccination as a general indication of well-being (Fig. 4A). OptiLAIV vaccinated mice showed no weight loss and no mortality while vaccination with LAIV resulted in weight loss 5 days postvaccination and 50% mortality (Fig. 4B).

This pronounced difference led us to explore the dose-range equivalence between LAIV and optiLAIV for the induction of adverse effects (Fig. S5A). We found that a LAIV dose of 10^3^ PFU caused equivalent body weight loss as a 10^5^ PFU dose of optiLAIV (Fig. S5B). Notably, one out of five animals vaccinated with 10^3^ PFU of LAIV lost more than 15% body weight and had to be sacrificed, while all optiLAIV vaccinated mice stayed above this threshold. In order to evaluate if the two vaccines might protect Stat1^−/−^ mice, the remaining animals were challenged with a lethal dose of A/Netherlands/602/2009 (H1N1) (Fig. S5C). We observed that all optiLAIV vaccinated mice survived with an overall mild weight loss, while LAIV vaccinated mice had a more pronounced weight loss, with one animal crossing the experimental threshold. Considering both vaccination and challenge, our results point toward a safety window of 100-fold between LAIV and optiLAIV doses.

In order to understand the differences in morbidity and mortality observed in Stat1^−/−^ mice, we quantified vaccine virus replication in both upper and lower respiratory tract. Compared to *wild-type* animals, Stat1^−/−^ animals have approximately 100-fold higher viral titers in both upper and lower respiratory tracts at 4 days postvaccination. Importantly, optiLAIV titers remained lower than LAIV titers in the lower respiratory tract, confirming its enhanced attenuated phenotype (Fig. 4C).

In conclusion, in the absence of a functional type I, II and III interferon response, optiLAIV vaccine virus safety profile was improved compared to LAIV.

### OptiLAIV induces the unfolded protein response *in vitro*.

The ability of NS1 to bind CPSF30 was thought to only antagonize the innate antiviral host response (46, 47). However, mutations of the CPSF30-binding domain of NS1 were recently described to modulate ER stress activation and the UPR through the XBP1 pathway (27). We introduced the same mutations in the NS1 of optiLAIV and hypothesized that the establishment of a host response, mediated by UPR, could explain the attenuated phenotype of optiLAIV. Indeed, the reduced viral replication of optiLAIV was STAT1-independent, pointing to a mechanism independent of the canonical interferon-mediated antiviral response.

Proving an involvement of ER stress *in vivo* is technically challenging. While knockout of the main UPR component, XBP1, is lethal during embryonic development, the fast turnover of this response largely narrows the window of detectable signal *in vivo*. In addition, there is high background signal from unspliced XBP1 mRNA present in uninfected cells. This technical hurdle was confirmed by analysis of two UPR-induced genes at vaccine virus peaks in neonatal mice respiratory tract (Fig. S4A). Thus, we turned to *in vitro* models to address our hypothesis.

**FIG 5:**
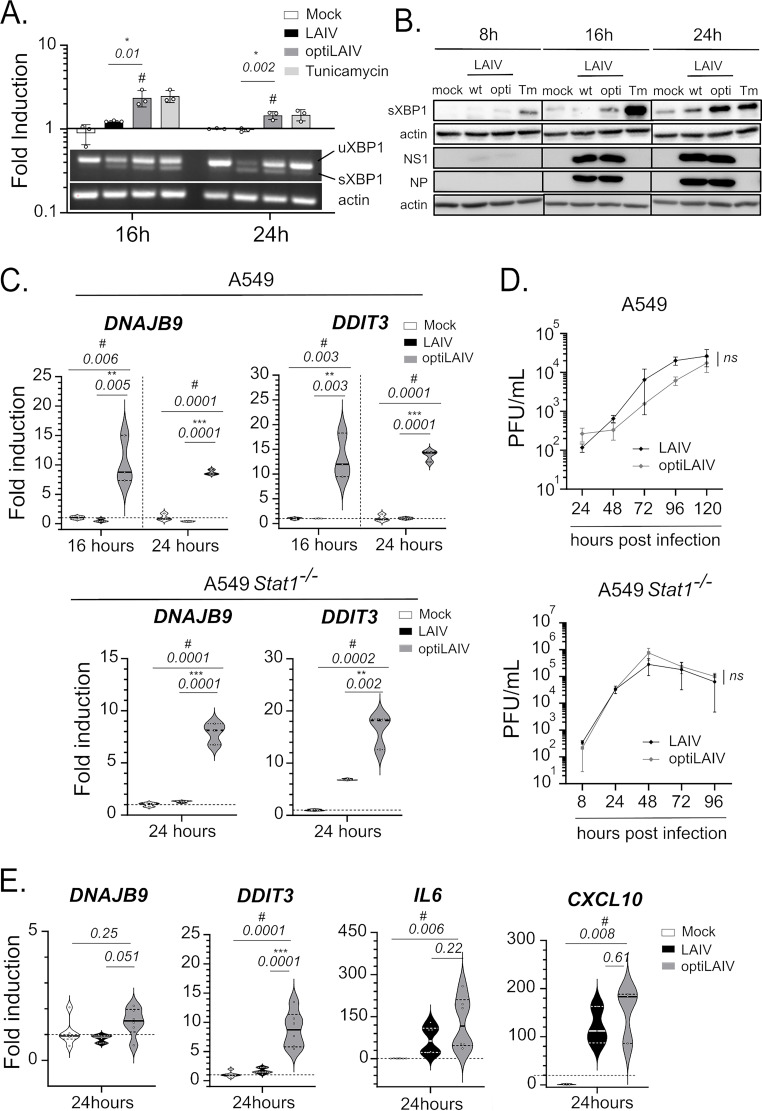
OptiLAIV NS1 allows unfolded protein response activation in human cell models. (A–B) A549 cells were infected at an MOI of 5 with LAIV or optiLAIV or treated with tunicamycin at 5 μg/mL. At 16 h and 24 h postinfection RT-PCR was performed from isolated RNA for the spliced form of XBP1 mRNA (A). In parallel at 8 h, 16 h and 24 h postinfection, lysates were analyzed by Western blot for expression of sXBP1, actin, NP and NS1 proteins (B). (C) A549*wt* cells (upper panel) or A549*Stat1^−/−^* cells (lower panel) were infected at an MOI of 5 with LAIV or optiLAIV and RT-qPCR for UPR-induced genes DNAJB9 and DDIT3 was performed in RNA lysates at 16 h and 24 h postinfection. (D) A549*wt* cells (upper panel) or A549*Stat1^−/−^* cells (lower panel) were infected at an MOI of 0.01 with either LAIV or optiLAIV. Supernatants were collected at indicated time postinfection and viral titers determined by plaque assay. (E) Primary human nasal epithelial cells (Mucilair) were infected at an MOI of 5 with LAIV or optiLAIV. Cells were lysed at 24 h postinfection and RT-qPCR for each respective gene was performed. The statistical significances between LAIV group and optiLAIV group were determined using one-way ANOVA with *post hoc* Tukey’s multiple-comparison test for panels A, C and E; two-way ANOVA with the Geisser-Greenhouse correction and *post hoc* Dunn’s multiple-comparison test for panel D. *, comparison between LAIV and optiLAIV; **^#^**: comparison between mock and optiLAIV; *ns* = nonsignificant. Graphs are representative of 3 independent experiments and indicate mean ± SD.

First, we asked if the lower CPSF30 binding ability of optiLAIV NS1 allowed the establishment of the UPR upon infection. We infected A549 human lung epithelial cells and evaluated the splicing of XBP1 mRNA by semiquantitative RT-PCR and the expression of the spliced form of XBP1 protein (sXBP1) by Western blot. sXBP1 mRNA levels in optiLAIV-infected A549 increased compared to LAIV (Fig. 5A). Accordingly, we observed that sXBP1 protein expression increased in a time-dependent manner after infection, an increase that was more pronounced in cells infected with optiLAIV (Fig. 5B). We also analyzed by RT-qPCR the induction of genes downstream of the transcription factor sXBP1, namely, *DDIT3* and *DNAJB9*. Compared to LAIV, expression of these two genes increased in cells infected with optiLAIV (Fig. 5C, upper panel), in line with previous published results (27). Of note, induction of UPR genes by optiLAIV was independent of Stat1 signaling (Fig. 5C, lower panel). This increase was also observed when we used a murine lung epithelial cell line (Fig. S4B) indicating that this phenomenon is species independent and could occur in our murine *in vivo* models. Additionally, viral replication was similar in all three cell models ruling out possible differences in replication kinetic as an explanation for the phenotype observed (Fig. 5D and Fig. S4C).

Next, we used a relevant 3D cell model of stratified primary human nasal epithelial cells grown in air-liquid interface. In line with the results obtained with immortalized epithelial cell lines, we observed an increased induction of UPR-induced genes in optiLAIV infected tissues compared to LAIV, and no differences in the induction of antiviral response genes or inflammatory cytokine genes (Fig. 5E). These cells would be the first in contact with LAIV virus in case of vaccination of human patients and the upregulation of DNAJB9 and DDIT3 in this model, corroborates our hypothesis of UPR as a potential optiLAIV attenuation mechanism.

## DISCUSSION

From a medical and immunological point of view the LAIV platform provides advantages over the inactivated vaccine, including easier, needle-free application, a broader immune response against IAV and a reduction of viral transmission due to mucosal immunity as demonstrated in animal models (48, 49). Regrettably, safety concerns exclude highly susceptible populations to IAV from benefiting from this vaccine platform.

In the present work, we modify LAIV protein NS1 in a targeted manner and characterize optiLAIV, a vaccine candidate with an increased attenuated profile without affecting its efficacy. Other groups described that an IAV with full NS1 deletion used as a single intranasal dose was well tolerated, safe, and able to induce neutralizing antibodies in three different clinical trials (20, 50, 51). Similarly, IAV vaccines expressing truncated NS1 were safe and protected both adult ferrets and aged mice (52, 53). These approaches, however, rely on blunt removal of the effector domain of NS1 affecting multiple host-pathogen interaction pathways. Additionally, efficacy and safety were not assessed against the current standard of care (LAIV). Our strategy to attenuate LAIV exploits a more targeted approach by performing five single amino acid substitutions in the currently licensed LAIV backbone and we characterized its safety profile in two relevant animal models that are representative of high-risk patient groups. Moreover, we compare efficacy and safety to the parental LAIV. Interestingly, a similar targeted approach mutated two key amino acids for CPSF30 binding (F103S/M106I) in the LAIV A/Ann Arbor/6/60 backbone; however, this strategy failed to revert the shutoff of the host response and did not increase LAIV safety in adult mice (54). Our data suggest that the additional three mutations in NS1 (K108R/D125E/D189G) are responsible for the accelerated clearance of optiLAIV from adult and neonatal mice.

Abrogation of NS1 binding to CPSF30 is largely associated with an increased expression of IFN-mediated genes (23–25, 35), however, optiLAIV’s attenuated phenotype was Stat1-independent, pointing to an alternative mechanism of attenuation. A thorough RNA-seq analysis performed in cells infected with weak NS1-CPSF30 binding influenza strains showed upregulation of UPR-induced genes (55). This observation led us to postulate that UPR activation during optiLAIV replication induces a self-adjuvanting mechanism contributing to its faster clearance without affecting its protective efficacy. In accordance, UPR activation was described in mice as the mechanism of action of AS03, a licensed adjuvant used in IIV formulation (32). Furthermore, upregulation of UPR-induced XBP1 after IIV administration in human patients was suggested as a molecular marker for seroconversion against the three vaccine strains present in the vaccine’s formulation (56).

One limitation of the present study is the lack of *in vivo* data linking UPR to the attenuated profile of optiLAIV. The transient nature of this pathway, together with the background noise from uninfected cells makes detection of ER stress *in vivo* challenging. On top, mice deficient for central elements of the ER stress sensing pathway are unviable (57). Still, our data encourages future studies to explore how UPR impacts the immune response to live replicating viral vaccines (58).

LAIV is approved for 2 to 49 years old immunocompetent patients. Our data provides the first attempt to characterize and improve it in a neonatal mouse model. We provide unprecedented evidence of LAIV cross-protection in a neonatal mouse model. Furthermore, the observed optiLAIV attenuation in this model could be used in combination with strategies to develop a universal vaccine against influenza. For example, matching internal influenza segments to circulating strains (59) and inducing immunity against conserved epitopes in the stem region of HA (60) could be strategies incorporated into the optiLAIV backbone. Generating an immunogenic yet safer vaccine could broaden LAIV application to high-risk patients. This is particularly interesting in children under 2 years old. In France alone, more than 28’000 children below the age of 2 were admitted to a hospital with an IAV infection for the period of 2011 to 2020 (61). While IIV is available for this target population, a needle-free applicable LAIV, which does not cause severe side effects, could increase the willingness of parents to vaccinate their children. Additionally, mathematical models predict better efficacy for live attenuated vaccines if vaccinees are children without previous exposure to circulating strains (62), which encourages LAIV implementation in this population in which protection from maternal antibodies start to wane (63, 64). This is specifically interesting since the first exposure to IAV is believed to imprint later immune responses (65). This bias could potentially be overcome by an early targeted exposure to H1 and H3 antigens in context of LAIV administration.

We also characterize for the first time LAIV in a a severely immunocompromised Stat1-deficient mouse model. While Stat1-deficient mice do not reproduce the full spectrum of phenotypes from a chemically induced immunosuppression, the absence of clinical signs after vaccination of optiLAIV in this model suggests that it could be safely used in target populations with milder immunosuppression such as organ transplant recipients or patients with other comorbidities. In fact, LAIV has already been shown safe without causing any adverse reactions in ferrets under a regimen of immunosuppressors (66). We hypothesize that the use of optiLAIV in immunocompromised patients would represent an additional safety measure to ensure the absence of side effects. Also, we showed in a preliminary proof-of-concept experiment that a higher dose of optiLAIV could be safely administered and achieved full protection. It would be of interest to further investigate the relation between optiLAIV vaccine dose and protection in clinically relevant animal models.

In summary, we propose here an optimized LAIV backbone, with an improved safety profile in adult, neonatal and immunocompromised hosts, while still protecting against lethal infection. Deriving from an already licensed vaccine, optiLAIV possesses the scalability to be produced at similar titers as the current LAIV, without major challenges. It would be interesting to study in more detail the immune responses triggered by this vaccine and its safety in higher mammal models before proceeding to clinical studies. In addition, the mechanism of attenuation remains to be fully characterized, although our data suggest a potential role of the UPR. Our approach opens the possibility to expand the applications of the current LAIV with the possibility of targeting high-risk populations in which IAV burden is still a serious problem.

## MATERIALS AND METHODS

### Materials availability.

All unique/stable reagents generated in this study are available from the Lead Contact with a completed Materials Transfer Agreement.

### Reagents.

Antibodies for immunoblotting include: mouse anti-actin HRP (Abcam number ab49900), mouse anti-Flag HRP (Sigma number A8592), rabbit anti-XBP1 (ThermoFisher number PA5-27650), rabbit anti-influenza NP (ThermoFisher number PA5-32242), rabbit anti-influenza NS1 (1-93) (kind gift from A. Garcìa-Sastre) and goat anti-rabbit IgG HRP (Sigma-Aldrich number A8275).

Antibodies and dyes for flow cytometry staining are detailed in Table 1.

**TABLE 1:** Antibodies and dyes for flow cytometry staining

Antibodies	Source	Clone	Cat. number
eBioscience Fixable Viability eFluor 780	Thermo Fischer	NA	65-0865-14
CD45 BUV395	BD biosciences	30-F11	564279
CD11b BV605	biolegend	M1/70	101237
CD11c Pecy7	Thermofischer	N418	25-0114-82
F4/80 PE e610	Thermofischer	BM8	61-4801-82
MHCII A700	Biolegend	M5/114.15.2	107622
Ly6G APC	Thermofischer	1A8-Ly6g	17-9668-82
Ly6C BV510	Biolegend	HK1.4	128033
CD68 BV421	Biolegend	FA-11	137017
NK1.1 PercPcy5.5	Thermofischer	PK136	45-5941-82
Siglec F PE	Thermofischer	1RNM44N	12-1702-82

All oligonucleotides (Table 2) were purchased from Microsynth (France).

**TABLE 2:** Oligonucleotides

Application	Name	Forward (5′ to 3′)	Reverse (5′ to 3′)
Mutation of CPSF30 binding site in	flank	TCGCTCTTCTGGGAGCAAAAGCAGGGTG	TCGCTCTTCTATTAGTAGAAACAAGGGTGTTTT
IAV NS1	mut1	CAAGGGACTGGAGCATGCTAATCCCCAGACAGAAAGTGGC	GCCACTTTCTGTCTGGGGATTAGCATGCTCCAGTCCCTTG
	mut2	GACCAGGCAATCATGGAGAAGAACATCATATTG	CAATATGATGTTCTTCTCCATGATTGCCTGGTC
	mut3	GGACTTGAATGGAATGGTAACACAGTTCGAGTC	GACTCGAACTGTGTTACCATTCCATTCAAGTCC
PCR	hXBP1	TTACGAGAGAAAACTCATGGCC	GGGTCCAAGTTGTCCAGAATGC
	βactin	GGGTCAGAAGGATTCCTATG	GGTCTCAAACATGATCTGGG
qPCR	hCXCL10	GGAACCTCCAGTCTCAGCACCA	AGACATCTCTTCTCACCCTTC
	hIL29	GCCCCCAAAAAGGAGTCCG	AGGTTCCCATCGGCCACATA
	hDDIT3	AGAACCAGGAAACGGAAACAGA	TCTCCTTCATGCGCTGCTTT
	hDNAJB9	TCTTAGGTGTGCCAAAATCGG	TGTCAGGGTGGTACTTCATGG
	mcxcl10	TTCACCATGTGCCATGCC	GAACTGACGAGCCTGAGCTAGG
	mddit3	GGAGGTCCTGTCCTCAGATGAA	GCTCCTCTGTCAGCCAAGCTAG
	mdnajb9	CTCCACAGTCAGTTTTCGTCTT	GGCCTTTTTGATTTGTCGCTC
	mccl5	TGCCCACGTCAAGGAGTATTTC	TCCTAGCTCATCTCCAAATAGTTGATG
	mtnfa-fwd	AGAAACACAAGATGCTGGGACAGT	CCTTTGCAGAACTCAGGAATGG
	mil6	TGAGATCTACTCGGCAAACCTAGTG	CTTCGTAGAGAACAACATAAGTCAGATACC

### Plasmids.

pCAGGS.Flag-CPSF30 and pCAGGS.Flag-ZsGreen. The plasmids of the LAIV strain A/AA/1960 ca (AA) were kindly provided by Peter Palese and Adolfo García-Sastre (Icahn School of Medicine at Mount Sinai, New York, NY, USA). pDZ plasmids contain a bidirectional expression cassette for a given influenza A gene segment and have been described previously (67). The AA NS viral segment was subcloned into a pDZ plasmid (68) to introduce the following mutations using site-directed mutagenesis: T333A, T334G (F103S), G344C (M106I), A349G, G350A (K108R), T401G (D125E) and A592G (D189G).

### Cell lines.

A549 and A549 STAT1^−/−^ (adenocarcinomic human alveolar basal epithelial cells, ATCC) were grown in DMEM/F12 (Dulbecco's Modified Eagle Medium: Nutrient Mixture F-12, Gibco) supplemented with 10% (vol/vol) heat-inactivated fetal bovine serum (Gibco) and 100 U/mL Pen/Strep. HEK 293T (human embryonic kidney, ATCC) and MDCK (Madin-Darby canine kidney, ATCC) cells were grown in DMEM (Dulbecco's Modified Eagle Medium, Gibco) supplemented with 10% (vol/vol) heat-inactivated fetal bovine serum (Gibco) and 100 U/mL Pen/Strep. LA4 (lung adenoma) cells were grown in DMEM/F12 (Dulbecco's Modified Eagle Medium: Nutrient Mixture F-12, Gibco) supplemented with 15% (vol/vol) heat-inactivated fetal bovine serum (Gibco) and 100 U/mL Pen/Strep. Human airway epithelia (MucilAir, Epithelix Sàrl, Switzerland) were maintained in Mucilair medium (Epithelix Sàrl, Switzerland) that was changed every 2 days. The total number of differentiated cells was estimated to be 200,000 cells per well. All cells were maintained at 37°C with 5% CO_2_ and 90% humidity.

### Animals.

All animal procedures were in accordance with federal regulations of the Bundesamt für Lebensmittelsicherheit und Veterenärwesen (BLV) Switzerland (Tierschutzgesetz) and approved by an institutional review board and the cantonal authorities (license number GE/105/19 and GE96). C57BL/6J mice and C57BL/6J Stat1^−/−^ (7 to 8 weeks of age) were purchased from Charles River Laboratories (France) and housed under SPF/BSL2 conditions. All animals were housed for 7 days to adjust to housing conditions under a strict 12 h light/dark cycle and fed *ad libitum* before entering an experiment or the breeding process. Breeding cages were checked three times a day during labor period and time of birth was recorded for each dam. Litters born in the same day were divided into equal groups and left with the lactating parent until weaning at 21 days of age. After weaning, males and females were separated in groups of 4 to 5 animals/cage.

### Viruses.

A/Netherlands/602/09 (H1N1) (mouse adapted after two passages in mice) (Neth602) was kindly provided by Krammer (Icahn School of Medicine at Mount Sinai, New York, NY, USA). A/Viet Nam/1203/2004 HALo (low pathogenic version) (H5N1) (VN1203) was rescued by reverse genetic as described previously (27).

Recombinant LAIV virus was produced using the eight-plasmid rescue system (68). The A/AA/1960 ca PB2, PB1, PA, NP, M and NSwt (GenBank: M23968.1) or NSmut pDZ plasmids and PR8 HA and NA pDZ plasmids were used to rescue the vaccine virus LAIV and optiLAIV. Briefly, HEK293T (6-well plate format, 1 × 10^6^ cells/well) were cotransfected in suspension, using Lipofectamine 2000 and Opti-MEM medium with 500 ng of each plasmid and incubated at 37°C. At 24 h posttransfection, supernatants were collected and used to infect fresh monolayers of MDCK cells (6-well plate format, 1 × 10^6^ cells/well) and incubated at 33°C with 5% CO_2_. After 48 h, unique viral clones were isolated after plaque assay on MDCK and grown either in embryonated eggs or in MDCK cells, as described before (69). Virus stocks were quantified by plaque assay in MDCK cells at 33°C and sequenced.

### Immunoprecipitation assay.

293T were transfected with 1 μg of pCAGGS.Flag-CPSF30 or with 0.5 μg of pCAGGS.Flag-ZsGreen using 2 μL/μg DNA of TransIT-LT1 (Mirus number Mir2304). 24 h posttransfection, cells were infected at an MOI of 5 with either LAIV or optiLAIV. Cells were lysed on ice after 16h in 300 μL IP lysis buffer (50 mM Tris HCl pH 7.5, 150 mM NaCl, 0.5% vol/vol NP-40, 5 mM EDTA with protease inhibitors [Pierce number 88266]). 50 μL of the cleared lysate were mixed 1:1 with protein lysis buffer (50 mM Tris HCl pH 6.8, 10% glycerol, 2% SDS, 0.1 M DTT and 0.1% bromophenol blue in H_2_O) to serve as whole-cell lysate control, while the remaining 200 μL was processed for immunoprecipitation over night at 4°C using anti-Flag M2 affinity gel (Sigma number A2220). Samples were then used in a Western blot.

### Viral growth kinetic.

Multicycle growth kinetics were carried out by infecting A549 cells (12-well plate format, 2 × 10^5^ cells/well, triplicates) with indicated viruses at a multiplicity of infection (MOI) of 0.01. After 45 min of viral adsorption at 33°C, supernatants were replaced by fresh infection medium supplemented with 0.1 μg/mL of N-tosyl-l-phenylalanine chloromethyl ketone (TPCK)-treated trypsin (Sigma number T1426) and plates were incubated at 33°C with 5% CO_2_. Supernatants were collected at the indicated times postinfection and viral titers were determined by plaque assay in MDCK cells.

### Infections.

For A549 and LA4, infections were carried out in a 12-well plate format (2 × 10^5^ cells/well) with indicated viruses at a multiplicity of infection (MOI) of 5. After 45 min of viral adsorption at 33°C, supernatants were replaced by fresh infection medium and incubated at 33°C with 5% CO_2_. Tunicamycin (Merck number T7765) was added to the culture medium at 5 μg/mL. At the indicated time point, cells were washed with PBS and either TRK lysis buffer (E.Z.N.A) or protein lysis buffer (TrisHCl 50 mM pH 6.8, glycerol 10%, SDS 2% and DTT 100 mM) was added to the wells and stored at −70°C until further analysis.

For human airway epithelia, cells were incubated with PBS supplemented with Ca^2+^ and Mg^2+^ (100 mg/L of CaCl_2_ and 100 mg/L of MgCl_2_−6H_2_0) for 45 min at 37°C, 5% CO_2_ and subsequently washed three times with PBS with Ca^2+^ and Mg^2+^. Cells were then inoculated in triplicates with LAIV or optiLAIV at MOI 5 for 3 h at 33°C, 5% CO_2_. Cells were then washed three times with PBS with Ca^2+^ and Mg^2+^ and then left at the air-liquid interface at 33°C with 5% CO_2_ and 90% humidity. At the indicated time point, cells were washed with PBS and TKR lysis buffer (E.Z.N.A) was added to the apical side. Lysates were collected and stored at −70°C until further analysis.

### RT-PCR and RT-qPCR.

Following infection, total cellular RNA was isolated using E.Z.N.A Total RNA kit 1 (Omega Bio-Tek number R6834-01) according to manufacturer’s instructions. cDNA was synthesized using M-MLV Reverse transcriptase (Promega number M170A) according to manufacturer’s instructions using 500 ng of RNA as starting material and 500 ng of oligo dT as primers.

To perform PCR, GoTaq G2 DNA polymerase (Promega number M784B) was used with 5× Green GoTaq reaction buffer (Promega number M791A). The primers were designed to flank the splicing site of XBP1 mRNA in order to amplify both the unspliced and spliced form. A PCR for β-actin was used as a control. PCR products were separated on a 3% agarose gel containing EtBr for visualization. Images were acquired using GelDoc XR+ System (Bio-Rad) and analyzed with Image Lab software (v4.1 from Bio-Rad).

To perform qPCR, 1 μL of cDNA was mixed with 10 μL of 2× KAPA SYBR FAST qPCR Master Mix-universal (KAPA Biosystems, USA), 1 μL of each respective forward (0.5 μM) and reverse (0.5 μM) primers in a final volume of 20 μL with RNase, DNase Free Molecular Biology Grade Water (Amimed, BioConcept, Switzerland). Quantitative PCR was performed following a thermal cycling protocol of an initial denaturation step at 95°C for 5 min, followed by 40 cycles of denaturation at 95°C for 30 s and annealing/extension at 60°C for 60s. After each reaction melting curves were determined for each primer set to confirm the correct amplification of the target gene.

### Western blots.

Cell lysates were sonicated and boiled for 5 min at 95°C. The same volume of lysates was loaded on a denaturing SDS-polyacrylamide gels, transferred to Polyvinylidene fluoride (PVDF) membranes (GE Healthcare), blocked with 5% skimmed milk in TBS containing 0,1% Tween 20 (Applichem) and incubated with the respective primary antibodies overnight at 4°C. Secondary anti-mouse or anti-rabbit conjugated to HRP were incubated for 1 h and developed using the WesternBright ECL-spray (Advansta).

### Plaque assays.

Monolayers of MDCK cells (6-well plate format, 1,5 × 10^6^ cells/well) were infected with 10-fold serial dilutions in PBS + 0,2% BSA of the correspondent virus for 45 min at 33°C. Supernatants were replaced by fresh overlay medium supplemented with 1 μg/mL of N-tosyl-l-phenylalanine chloromethyl ketone (TPCK)-treated trypsin (Sigma) and 0,6% purified agar (Oxoid) and plates were incubated at 33°C 5% CO_2_ (70). After 48 h, cells were fixed for 1 h at RT with 4% paraformaldehyde (PFA) and the overlays were removed. Cells were stained using a solution of 16% Metanol and crystal violet and plaques were counted visually.

### Flow cytometry.

Lungs and snouts were cut into small pieces and digested in RPMI containing 1 mg/mL collagenase IV (cat. number LS004189, Worthington Biochemical Corporation), 40 mg/mL DNase I, and 2% FCS for 30 min at 37°C. Any tissue remaining after 30 min was further digested with 1 mg/mL collagenase D (cat. number 11088882001, Roche), and 40 mg/mL DNase I and 1% of FCS for 20 min at 37°C. The reaction was stopped by addition of 5 mmol/L EDTA and 10% BSA. Samples were further disaggregated through a 70-mm cell strainer and blocked with anti-CD16/32 (cat. number 14-0161-86, Invitrogen). Single-cell suspensions were counted and stained with antibodies as previously described (71). Intracellular staining with anti-CD68 was done using the Intracellular Fixation and Permeabilization buffer set (Thermo Fisher): Fixation/permeabilization concentrate (cat. number 00-5123-43) and Diluent (cat. number 00-5223-56), Permeabilization Buffer 10 × (cat. number 00-8333-56). Cells were acquired on a Fortessa and analyzed using FlowJo software using the gating strategy depicted in Fig. S2A.

### Animal vaccination.

For neonatal mice, at 7 days postbirth, pups were weighed and marked. All animals over 3 g in weight were inoculated with 5 μL of the indicated dose of vaccine diluted in cold-PBS via the intranasal route.

For adult mice, 8 weeks-old females were weighed, marked and injected intraperitoneally with 10 μL/g of ketamin/xylazine (100 mg/kg and 5 mg/kg, respectively) diluted in sterile PBS. Upon reaching deep anesthesia, mice were inoculated with the respective doses of each vaccine in 20 μL PBS via the intranasal route.

To assess vaccine susceptibility, body weight was monitored for 14 days after vaccination. For neonatal mice, the cutoff threshold was determined as the interval from the difference between the average weight of the mock-vaccinated group and the lightest mouse of that group (biological variation of the pups’ weight), times two, calculated for each day postvaccination. For adults, a 15% body weight loss from the initial weight was used.

To assess virus replication, 7 days-old mice or 8 weeks-old females were vaccinated with 1 × 10^5^ PFU of either vaccine and at the indicated day postvaccination animals were euthanized by decapitation (pups) or controlled CO_2_ exposure (adults). Snouts and lungs were harvested immediately after euthanasia using sterile tools in 1 mL cold-PBS and homogenized twice with ¼’’ ceramic grinding balls (MPBio, USA) using a Bead Blaster 24 (Benchmark Scientific, USA) with a speed setting of 6 m/s for 30 s with a 30 s break on ice between each cycle. Samples were centrifuged at 10,000 × *g* for 10 min at 4°C. Supernatants were collected and frozen at −70°C to be used in plaque assays. Pellets were resuspended in 1 mL TRIzol (Invitrogen) and frozen at −70°C for further RNA extraction.

To assess antibodies response, blood samples were harvested on the respective days by lateral tail-vein bleeding. Briefly, mice were placed under a plastic restrainer and an orthogonal incision was made on the left vein of the tail. Blood was collected in 1,5 mL polystyrene tubes and incubated at room temperature for 30 min to 1 h to allow coagulation. Samples were centrifuged at 10,000 × *g* for 5 min at room temperature and supernatants collected into fresh tubes and stored at −20°C to be used in microneutralization assays.

### Animal challenge.

At the indicated day, vaccinated mice were injected intraperitoneally with 10 μL/g of ketamin/xylazine (100 mg/kg and 5 mg/kg, respectively) diluted in sterile PBS. Upon reaching deep anesthesia, mice were challenged with 20 μL of 10 × LD_50_ of A/Netherlands/2009 (H1N1) or 20 × LD_50_ of A/VietNam/1203/2004 (H5N1) HALo (low pathogenic version) in cold-PBS via the intranasal route. Body weight was monitored for 14 days postchallenge and upon reaching experimental or humane endpoints (85% of initial body weight) animals were euthanized using controlled CO_2_ exposure.

### Statistics.

Statistical analysis was performed using GraphPad Prism 8 and statistical tests applied are indicated in each respective figure legend.

### Data availability.

The data generated and analyzed during this study are available from the corresponding author upon reasonable request.
