# Supplementary material for: Optimizing the Live Attenuated Influenza A Vaccine Backbone for High-Risk Patient Groups
Source: J Virol. 2022 Oct 3;96(20):e00871-22. doi: 10.1128/jvi.00871-22 (PMC9599596; doi:10.1128/jvi.00871-22)
Supplement: Supplemental file 1 — Fig. S1 to S5. Download jvi.00871-22-s0001.pdf, PDF file, 1.9 MB [file jvi.00871-22-s0001.pdf]

Figure S1

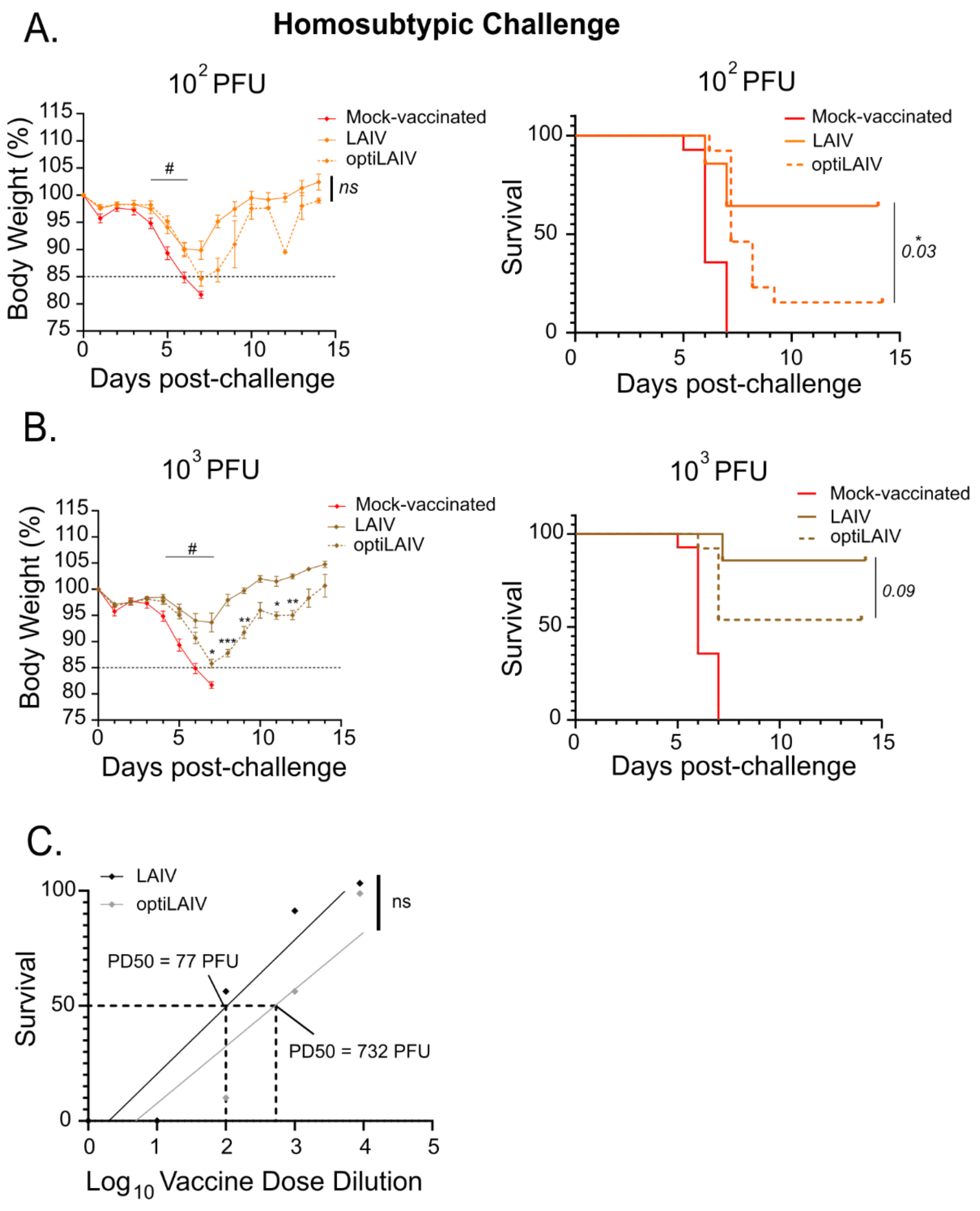

Figure S1: LAIV and optiLAIV body weight loss and survival in adult mice after challenge.

(A – B) Female 8-weeks-oldmice vaccinated with 10^2 PFU (A) or 10^3 PFU (B) of either LAIV or optiLAIV were challenged at day 21 post-vaccination with 20 PFU ($10\times LD_{50}$) of A/Netherlands/602/2009 (H1N1) in 20 µL PBS under anaesthesia (n=14 per group).

Body weight (left panels) and survival (right panels) were monitored for 14 days post-challenge.

(C) PD_{50} was calculated according to Reed & Muench. Linear regression was used to determine statistical significance between the two PD_{50}.

The statistical significances between LAIV group and optiLAIV group were determined using two-way ANOVA with the Geisser-Greenhouse correction and post-hoc Dunn's multiple comparisons test for panels A and B and Mantel-Cox test for survival curve. *: comparison between LAIV and optiLAIV; $^{\#}$: comparison between mock and optiLAIV; *ns* = non-significant. Graphs are representative of 3 independent experiments and indicate mean $\pm$ SEM.

Figure S2

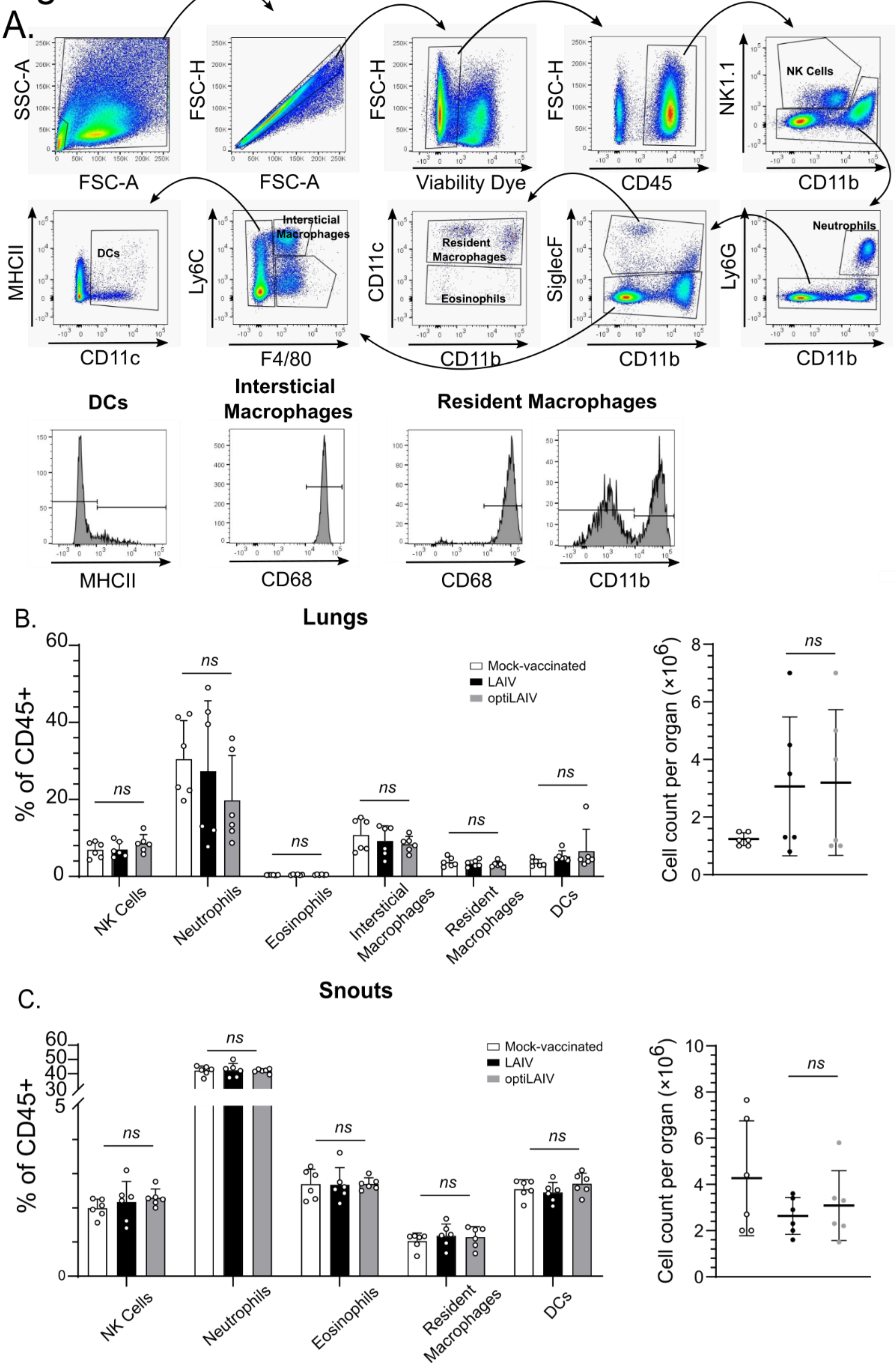

Figure S2: Cell infiltrates after LAIV administration in adult mice respiratory tract .

(A) Gating strategy (B-C) Female 8-weeks-old mice were vaccinated intranasally under anaesthesia with 10^5 PFU LAIV or optiLAIV in 25 μL and at 4 days post-vaccination (B) lungs and (C) snouts were collected, homogenized and cells were stained for different surface markers to distinguish between different immune cell populations (left panels) and total cell number was determined counting cells manually (left panels). The statistical significances between LAIV group and optiLAIV group were determined using two-way ANOVA and post-hoc Tukey's multiple comparisons test for left panels and one-way ANOVA and post-hoc Tukey's multiple comparisons test for right panels. *ns* = non-significant. Graphs are representative of 2 independent experiments and indicate mean ± SD.

Figure S3

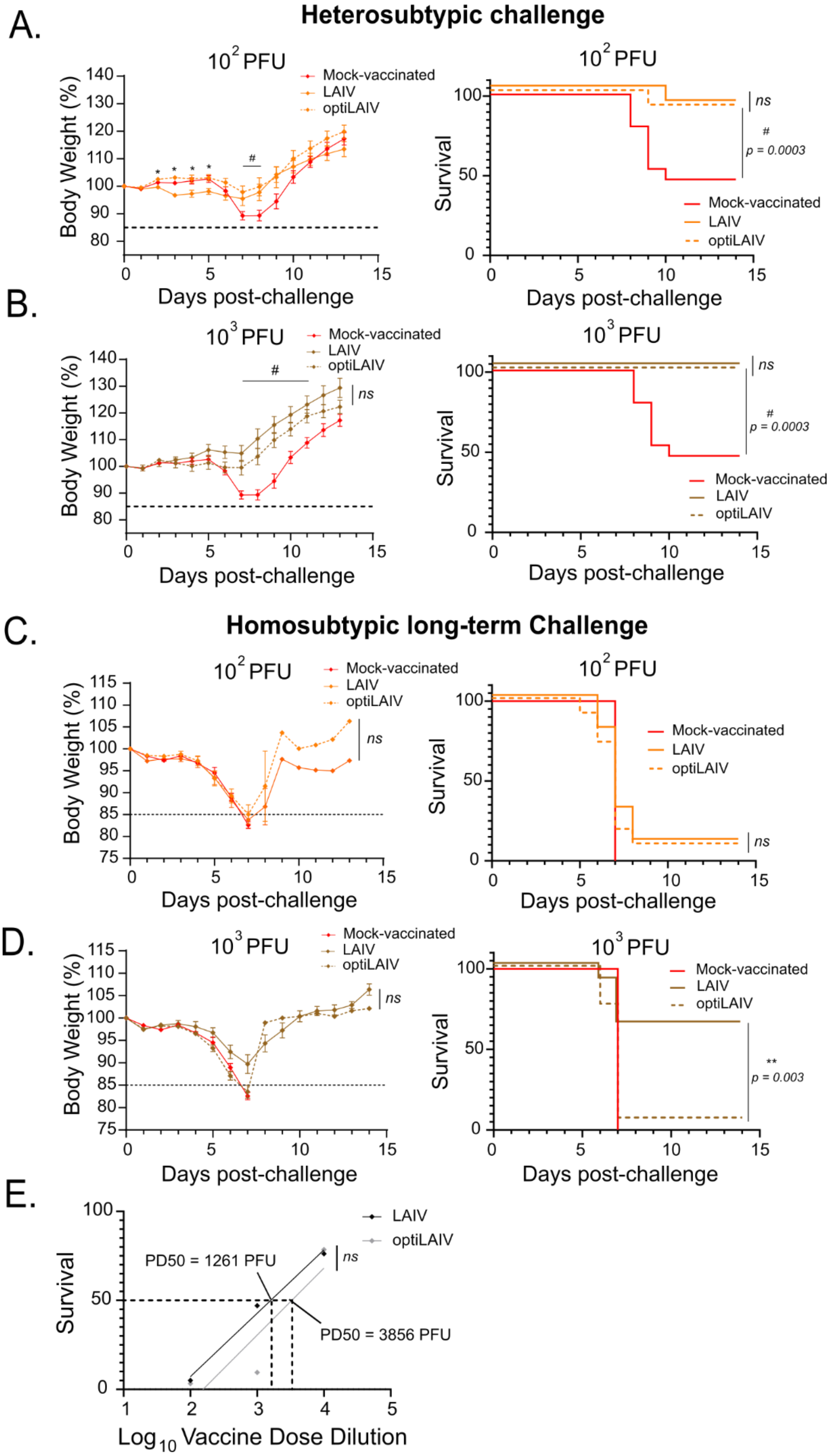
A.
Heterosubtypic challenge
10^2 PFU
Mock-vaccinated
LAIV
optiLAIV
Body Weight (%)
Days post-challenge
Survival
ns
#
p = 0.0003
B.
10^3 PFU
C.
Homosubtypic long-term Challenge
D.
**
p = 0.003
E.
LAIV
optiLAIV
PD50 = 1261 PFU
PD50 = 3856 PFU
Log10 Vaccine Dose Dilution

Figure S3: Body weight loss and survival after challenge in neonatal mice previously vaccinated with LAIV and optiLAIV.

(A – B) Seven-days-old mice were vaccinated intranasally with 10^2 PFU (A) or 10^3 PFU (B) of LAIV or optiLAIV in 5 μL PBS. At day 21 post-vaccination, mice were challenged with 10^3 PFU ($20\times LD_{50}$) of A/Vietnam/1203/2004 (H5N1) in 20 μL PBS under anaesthesia (n=11 per group). Body weight (left panels) and survival (right panels) were monitored for 14 days post-challenge.

(C – D) Seven-days-old mice were vaccinated intranasally with 10^2 PFU (C) or 10^3 PFU (D) of LAIV or optiLAIV in 5 μL PBS. At day 49 post-vaccination, mice were challenged with 20 PFU ($10\times LD_{50}$) of A/Netherlands/602/2009 (H1N1) in 20 μL PBS under anaesthesia (n=11-17 per group). Body weight (left panels) and survival (right panels) were monitored for 14 days post-challenge.

(E) PD_{50} was calculated according to Reed & Muench. Linear regression was used to determine statistical significance between the two PD_{50}.

The statistical significances between LAIV group and optiLAIV group were determined using two-way ANOVA with the Geisser-Greenhouse correction and post-hoc Dunn's multiple comparisons test for panels A-D and Mantel-Cox test for survival curve. *: comparison between LAIV and optiLAIV; $^{\#}$: comparison between mock and optiLAIV ; *ns* = non-significant. Graphs are representative of 2 independent experiments and indicate mean ± SEM.

Figure S4

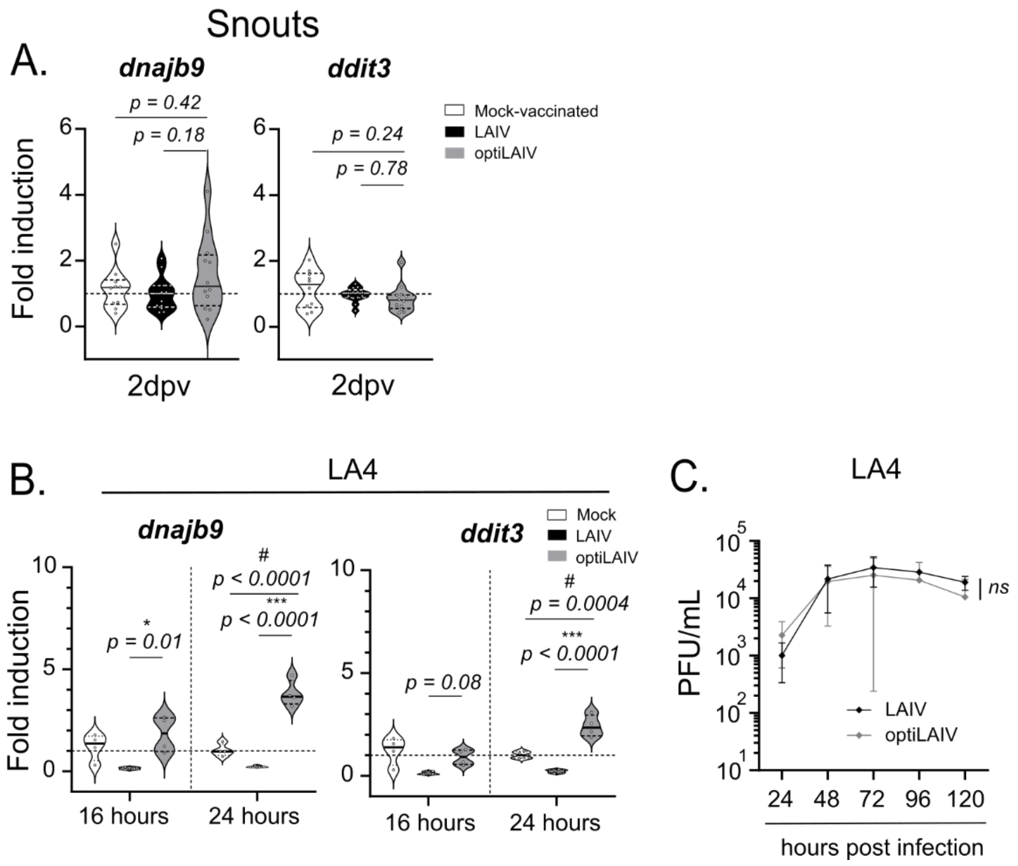

Figure S4: UPR-induced genes after LAIV and optiLAIV vaccination in neonatal snouts and murine lung cell lines.

(A) Seven-days-old mice(n=11-12) were vaccinated intranasally with 10^5 PFU of LAIV or optiLAIV in 5 μL PBS. At 2 days post-vaccination snouts were harvested and RT-qPCR performed in isolated RNA for UPR-induced genes *Dnajb9* and *Ddit3*.

(B) LA4 cells were infected at a MOI of 5 with LAIV or optiLAIV and RT-qPCR for UPR-induced genes *Dnajb9* and *Ddit3* was performed in RNA lysates at 16h and 24h post infection.

(C) LA4 cells were infected at a MOI of 0.01 with either LAIV or optiLAIV. Supernatants were collected at indicated time post infection and viral titers determined by plaque assay.

The statistical significances between LAIV group and optiLAIV group were determined using one-way ANOVA and post-hoc Tukey's multiple comparisons test in panels A and B; two-way ANOVA with the Geisser-Greenhouse correction and post-hoc Dunn's multiple comparisons

test for panel C. *: comparison between LAIV and optiLAIV; #: comparison between mock and optiLAIV; *ns* = non-significant. Graphs are representative of 2-3 independent experiments and indicate mean ± SD.

Figure S5

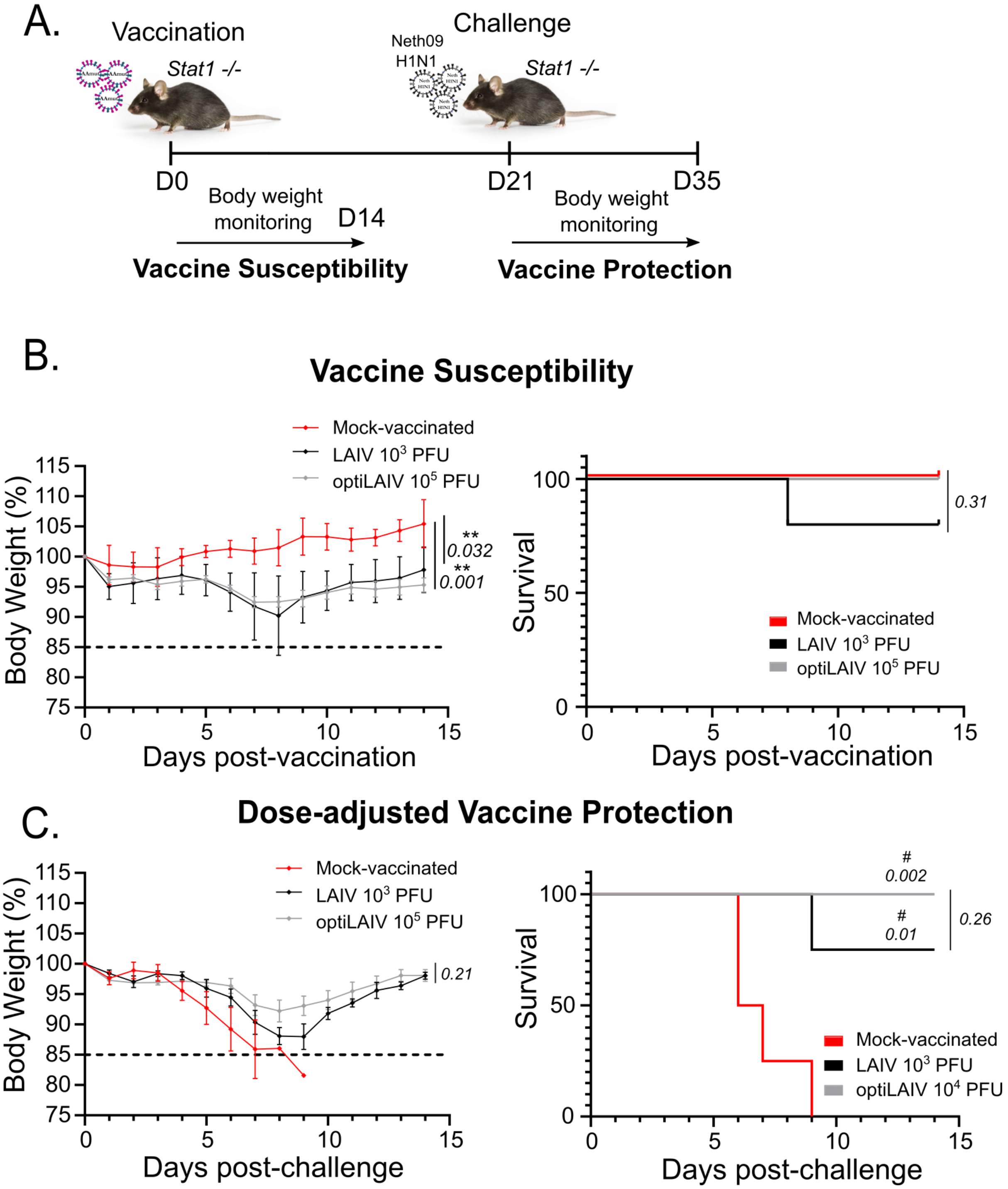

Figure S5. optiLAIV protects stat1-/- mice against homosubtypic challenge.

(A) Scheme of mouse immunization and challenge.

(B) Eight-weeks-old Stat1$^{-/-}$ mice were vaccinated intranasally under anaesthesia with 103 PFU LAIV or 105 PFU optiLAIV in 25 µL and body weight (left panel) and survival (right panel) were monitored for 14 days post-vaccination (n=4 for mock-vaccinated group; n=5 per vaccinated group).

(C) Female 8-weeks-old mice vaccinated with 10^3 PFU of LAIV or 10^5 PFU of optiLAIV were challenged at day 21 post-vaccination with 20 PFU (10×LD50) of mouse adapted A/Netherlands/602/2009 (H1N1) in 25 uµL PBS under light anaesthesia (n=4 in mock-vaccinated and LAIV group and n=5 in optiLAIV group). Body weight (left panel) and survival (right panel) were monitored for 14 days post-challenge. The statistical significances between LAIV group and optiLAIV group were determined using; two-way ANOVA with the Geisser-Greenhouse correction and post-hoc Dunn's multiple comparisons and Mantel-Cox long-rank test for survival curve. p-values are indicated in the figure. Graphs indicate mean ± SEM . Black dotted line represents 15% body weight loss cut-off.
